# Multifocal Pneumonia Amidst the Global COVID-19 Pandemic: A Case of Daptomycin-Induced Eosinophilic Pneumonia

**DOI:** 10.7759/cureus.16002

**Published:** 2021-06-28

**Authors:** Abi Watts, Christian C Toquica Gahona, Kavin Raj

**Affiliations:** 1 Internal Medicine, Saint Peter's University Hospital, New Brunswick, USA; 2 Internal Medicine, Saint Peter’s University Hospital, New Brunswick, USA

**Keywords:** covid-19, multifocal pneumonia, viral pneumonia, daptomycin induced eosinophilic pneumonia, eosinophilic pneumonia

## Abstract

Multifocal pneumonia amidst this global pandemic is often attributed to COVID-19, resulting in missed diagnosis of other potentially fatal illnesses such as eosinophilic pneumonia. Eosinophilic pneumonia is often associated with antibiotics and non-steroidal anti-inflammatory drugs. A 65-year-old male presented to the emergency department for a four-day history of fatigue, cough, and worsening dyspnea; CT thorax showed extensive multifocal pneumonia, and COVID-19 was suspected. COVID-19 testing using reverse transcription polymerase chain reaction was negative, and complete blood count revealed peripheral eosinophilia, which is not expected in COVID-19. The patient was being treated concomitantly with daptomycin and ceftaroline for septic arthritis and methicillin-resistant *Staphylococcus aureus* bacteremia. We reconsidered our initial diagnosis and held daptomycin, after which the patient started to improve. Due to hypoxia, steroids were added, which resulted in a dramatic improvement of the patient's symptoms. Daptomycin can have toxic effects, resulting in the accumulation of eosinophils in the lung parenchyma. Symptoms usually arise by the third week and include dyspnea, peripheral eosinophilia, and infiltrates involving the outer one-third of the lung fields. FDA drug safety guidance helped to establish this diagnosis. The treatment options include the removal of offending agents and steroids in severe cases.

## Introduction

Multifocal pneumonia amidst this global pandemic is often attributed to COVID-19, resulting in missed diagnosis of other serious illnesses, including eosinophilic pneumonia, which is rare [1]. The most common entities associated with eosinophilic pneumonia are antibiotics and non-steroidal anti-inflammatory drugs (NSAIDs), although other chemicals have also been reported [2]. We present a similar case of multifocal pneumonia, initially diagnosed as COVID-19 pneumonia but later identified as daptomycin-induced eosinophilic pneumonia.

This article was previously presented as a poster at the 15th Annual Internal Medicine Residency Research Day at Saint Peter’s University Hospital on May 27, 2021.

## Case presentation

**Figure 1:**
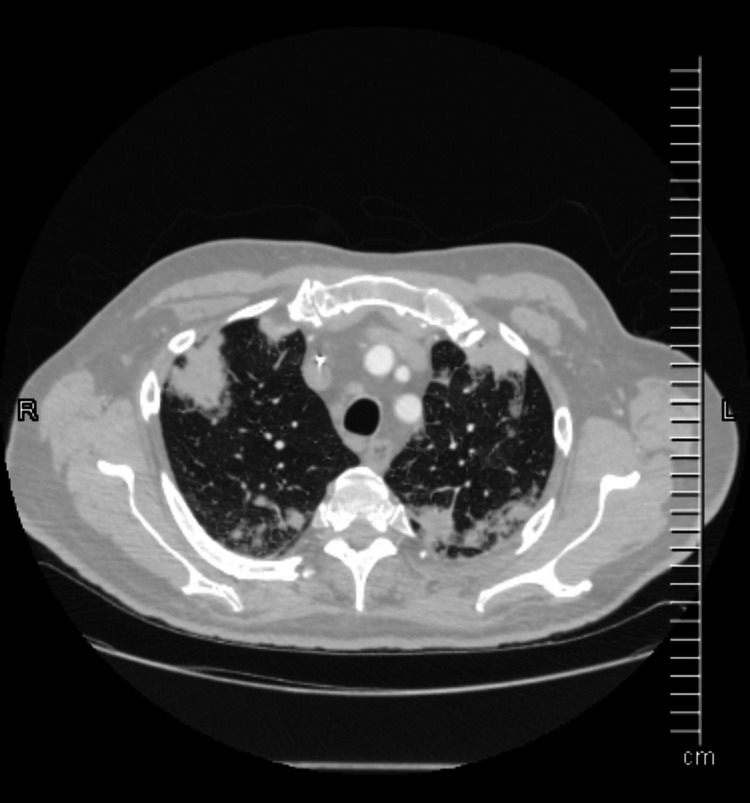
CT scan at presentation.

A 65-year-old male with a past medical history of recently recovered COVID-19 pneumonia, uncontrolled diabetes, basal cell carcinoma status after excision surgery complicated by non-healing wound, methicillin-resistant *Staphylococcus aureus* (MRSA) bacteremia, and septic arthritis presented for evaluation of fatigue, cough, and worsening dyspnea over the last four days. No reports of associated fever, chills, GI symptoms, and smoking/vaping history were present. He completed three out of six weeks of IV antibiotics with daptomycin (8 mg/kg/d) and ceftaroline for MRSA bacteremia and septic arthritis. He was tachypneic and hypoxic at presentation, with reduced breath sounds and diffuse rhonchi bilaterally on auscultation. Leukocytosis of 16.9 was present with the absolute lymphocyte and eosinophil count of 0.9 and 1.0, respectively. Elevation of inflammatory markers was present, and CT thorax showed extensive multifocal pneumonia (Figure 1).

Given his recent history of COVID-19 pneumonia, repeat infection of COVID-19 was suspected; however, polymerase chain reaction for COVID-19 was negative twice, and the patient's subsequent blood counts showed peripheral eosinophilia, the highest of which was 2.48 or 27% of total white blood cells count, which is not expected with COVID-19. Given the elevated eosinophil count and extensive multifocal consolidations, suspicion for daptomycin-induced eosinophilic pneumonia was high, and daptomycin was held. The patient had clinical improvement in his symptoms within 48 h of removing the inciting agent, daptomycin, in this case. Given his severe symptoms and hypoxia, he was treated with a tapering regimen of corticosteroids. Broncho-alveolar lavage was not attempted, given the clinical improvement with the above measures and to limit exposure amidst the pandemic.

## Discussion

**Figure 2:**
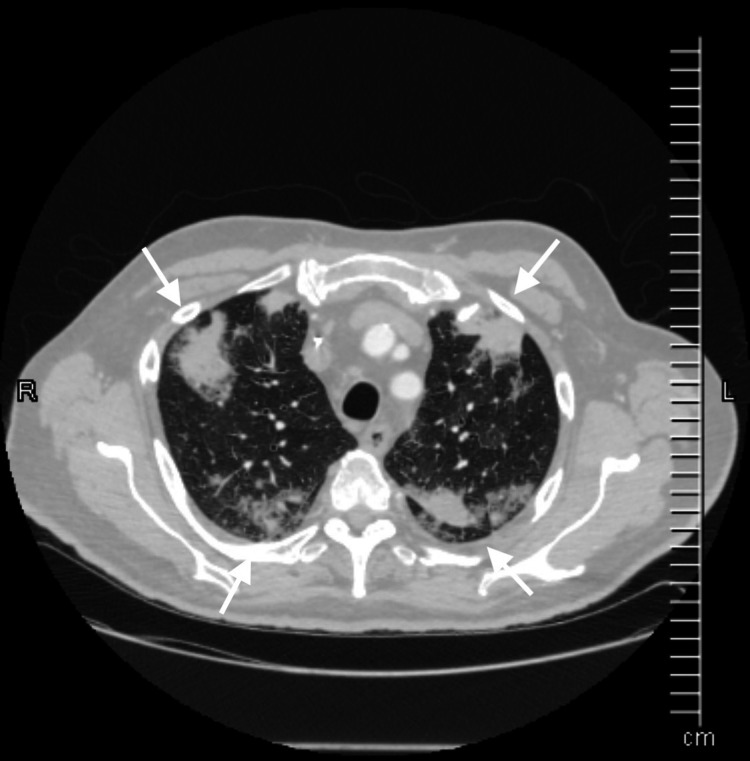
CT showing involvement of outer one-third of lung zones.

Daptomycin-induced eosinophilic pneumonia is a rare but a potentially fatal illness caused by the accumulation of eosinophils in the lung parenchyma with or without evidence of peripheral eosinophilia [1]. Long-term administration of this cyclic lipopeptide antibiotic, particularly in the elderly male population with renal dysfunction, can result in pulmonary toxicity even if dosed renally [1,3,4]. Symptoms of pulmonary toxicity appear by the third week into the daptomycin therapy and include difficulty in breathing, peripheral eosinophilia, and infiltrates involving the outer one-third of the lung fields being observed on chest imaging (Figure 2) [1,5]. 

Pulmonary toxicity occurs due to the accumulation of daptomycin near the epithelial alveolar surface, which alters lipid integrity and stimulates inflammatory response, resulting in antigen detection by alveolar macrophages and activation of TH-2 cells. Activation of TH-2 cells results in the release of interleukin 5, which causes increased production and migration of eosinophils into the lung parenchyma. Alveolar macrophages additionally also release eotaxin, which selectively induces chemotaxis of eosinophils into the lung parenchyma. Daptomycin-induced eosinophilic pneumonia is diagnosed with FDA drug safety guidance (Table 1) or Solomon and Schwartz criteria [2,6]. 

**Table 1:** FDA guideline for daptomycin-induced eosinophilic pneumonia.

Clinical features	Definite	Probable	Possible
Concurrent exposure to daptomycin	✔︎	✔︎	✔︎
Clinical improvement after withdrawal of daptomycin	✔︎	✔︎	✔︎
New infiltrates on chest X-ray/CT	✔︎	✔︎	✔︎
Dyspnea with increased oxygen requirements/mechanical ventilation	✔︎	✔︎	✘
Bronchoalveolar lavage with eosinophils	✔︎ (>25%)	✔︎ (<25%)	✘
Fever	✔︎	✘	✘

**Figure 3:**
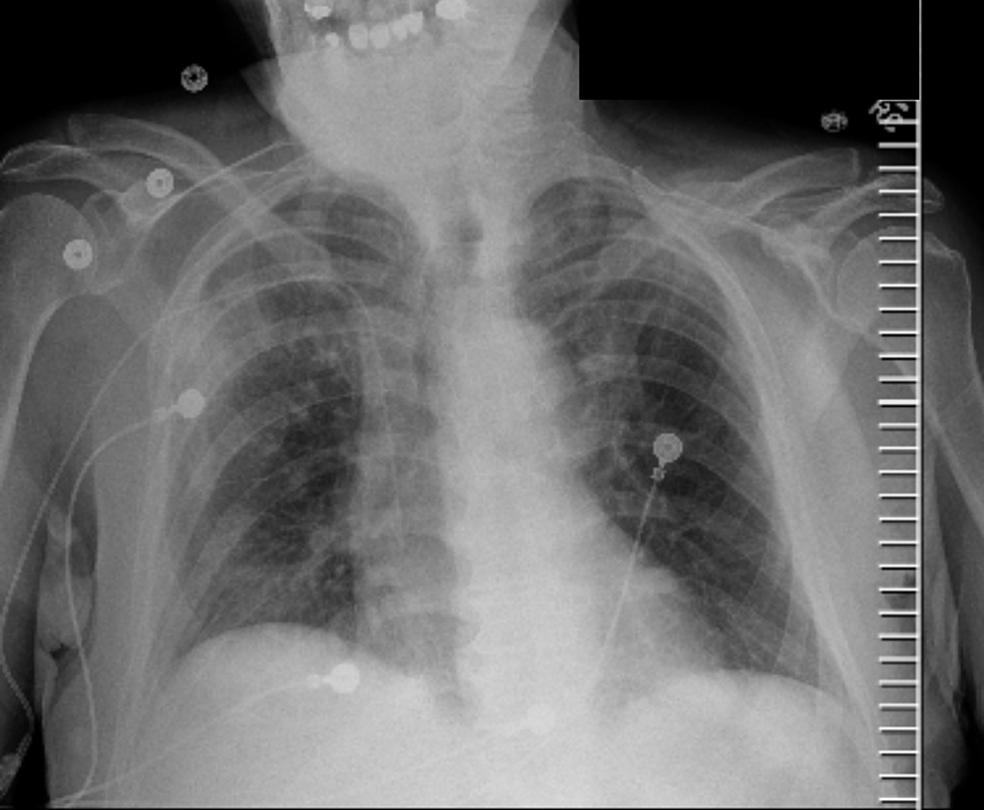
X-ray at presentation.

**Figure 4:**
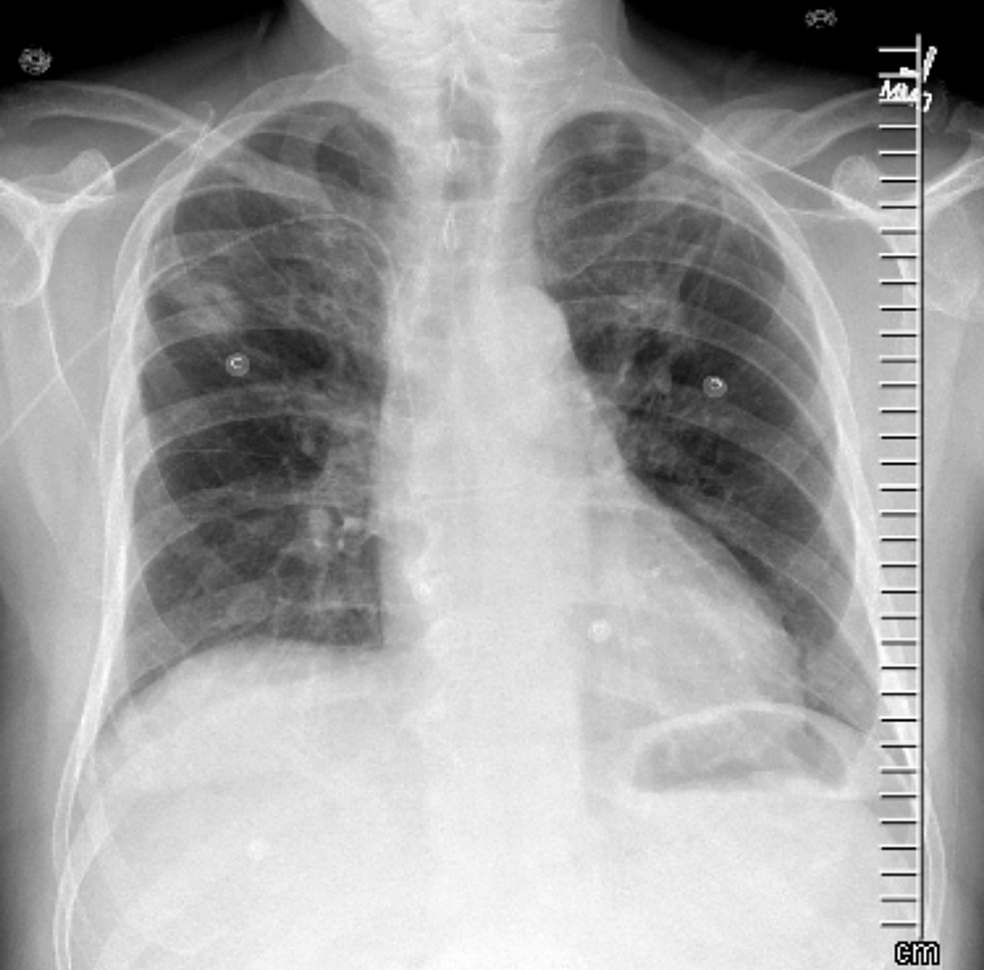
X-ray upon discharge.

Solomon and Schwartz included lung biopsy as a diagnostic criterion and advised ruling out fungal and parasitic infection and recurrence of eosinophilic pneumonia upon rechallenge in addition to the FDA guidelines. Lung biopsy is indicated only in a questionable case with treatment failures [4], and rechallenge is discouraged as it can be fatal. Management typically involves removal of daptomycin and tapering corticosteroids. Corticosteroids accelerate intracellular signaling in eosinophilic apoptosis, resulting in improvement in clinical symptoms and radiological abnormalities (Figures 3, 4).

The typical regimen includes prednisone 40-60 mg daily, tapered over two weeks; longer courses do not offer any additional benefit in clinical or radiological recovery. Symptomatic improvement usually occurs one to seven days after initial management, and most cases resolve; however, a few reports of incomplete recovery and chronic steroid dependence are present in the literature [1,7]. 

## Conclusions

Eosinophilic pneumonia should be kept in mind when treating multifocal pneumonia. It is crucial when known offending agents, such as antibiotics and NSAIDs, are present. FDA drug safety guidance or Solomon and Schwartz criteria can be used to establish the diagnosis. Prompt treatment with the removal of offending agents and steroids is necessary in severe cases.
